# Computed Tomography of the Spine: Systematic Review on Acquisition and Reconstruction Techniques to Reduce Radiation Dose

**DOI:** 10.1007/s00062-022-01227-1

**Published:** 2022-11-22

**Authors:** Michael Dieckmeyer, Nico Sollmann, Karina Kupfer, Maximilian T. Löffler, Karolin J. Paprottka, Jan S. Kirschke, Thomas Baum

**Affiliations:** 1 Department of Diagnostic and Interventional Neuroradiology, School of Medicine, Klinikum rechts der Isar, Technical University of Munich, Munich, Germany; 2 TUM-Neuroimaging Center, Klinikum rechts der Isar, Technical University of Munich, Munich, Germany; 3 Department of Diagnostic and Interventional Radiology, University Hospital Ulm, Ulm, Germany; 4 Department of Diagnostic and Interventional Radiology, University Medical Center Freiburg, Freiburg im Breisgau, Germany

**Keywords:** Multi-detector computed tomography, Dose reduction, Low dose, Image reconstruction, Image acquisition

## Abstract

The introduction of the first whole-body CT scanner in 1974 marked the beginning of cross-sectional spine imaging. In the last decades, the technological advancement, increasing availability and clinical success of CT led to a rapidly growing number of CT examinations, also of the spine. After initially being primarily used for trauma evaluation, new indications continued to emerge, such as assessment of vertebral fractures or degenerative spine disease, preoperative and postoperative evaluation, or CT-guided interventions at the spine; however, improvements in patient management and clinical outcomes come along with higher radiation exposure, which increases the risk for secondary malignancies. Therefore, technical developments in CT acquisition and reconstruction must always include efforts to reduce the radiation dose. But how exactly can the dose be reduced? What amount of dose reduction can be achieved without compromising the clinical value of spinal CT examinations and what can be expected from the rising stars in CT technology: artificial intelligence and photon counting CT? In this article, we try to answer these questions by systematically reviewing dose reduction techniques with respect to the major clinical indications of spinal CT. Furthermore, we take a concise look on the dose reduction potential of future developments in CT hardware and software.

## Key Points

- Spinal CT has high potential for dose reduction of 50% or more for the majority of clinical applications.
- Options and limitations of dose reduction are highly dependent on the clinical indications and application-specific techniques can further increase the achievable dose reduction.
- Additional dose reduction can be expected from the clinical transition of artificial intelligence and photon counting CT in the upcoming years.

## Introduction

The number of computed tomography (CT) examinations performed has been on the rise for decades [1–3]. Increases in clinical application are related to technical developments, wider availability, and physician and patient demands [1, 2]. CT is at the forefront of imaging for multiple purposes, spanning from regular oncologic staging to acute imaging in the emergency trauma setting, contributing significantly to accurate diagnosis, optimized patient management, and improved treatment; however, the use of CT is inherently accompanied by exposure to ionizing radiation, which may cause radiation-induced malignancies [4, 5]. More specifically, it is assumed that about 2% of future cancer cases will be attributable to current application of imaging techniques [3, 6]. Thus, a general principle is to keep radiation exposure as low as reasonably achievable (ALARA principle) [7, 8]; however, in daily clinical routine, CT-related radiation exposure still varies considerably within and across institutions, given that well-defined and ubiquitous reference standards are frequently missing [9, 10]. One relevant aspect is that general recommendations are hard to determine considering the various scanner models and technologies, which may exert an impact on radiation exposure during scanning of different body regions.

CT examinations of the spine are performed for different indications including fracture detection and trauma evaluation, assessment of degenerative changes, postoperative complications, and guidance of interventional procedures, such as periradicular infiltration (PRI) [11–13]. Particularly in musculoskeletal and neuroradiology departments, spinal CT constitutes a large proportion of the daily workload. Evidently, the most effective way to reduce CT-related radiation exposure is to use the technique only when the clinical value outweighs the risks and costs. Aside from that, various developments have emerged on both the acquisition and the reconstruction sides to achieve an optimized trade-off between image quality (IQ) and radiation exposure [14, 15]. Among others, dose reduction techniques include shielding of radiosensitive organs [16], beam-shaping filters [17] and, most importantly, the optimization of acquisition parameters, including tube voltage (kV), tube current, voxel size and slice thickness. Tube current is expressed either directly (as mA) or indirectly in terms of tube current-time product (as mAs). Different parameter combinations can lead to entirely different IQ at the same radiation dose.

In clinical CT examinations, radiation exposure is normally controlled via tube current modulation, which nowadays is usually achieved by means of automatic exposure control (AEC) [18, 19]. By adjusting the tube current to the patient’s habitus in the axial plane and along the z‑axis, a considerable dose reduction can be achieved. Tube current reduction results in a decreased patient dose, as the amount of generated X‑ray photons is directly proportional to the tube current [20]; however, image noise increases exponentially, mostly driven by Poisson noise. Exponential noise increase can be avoided by pulse-width modulation of tube current or X‑ray flux. This technique, termed sparse-sampling CT, reduces projections generated during a 360° gantry rotation while the dose for each individual projection remains constant. Technical implementations are challenging as they require high voltage fast switching electrical elements or fast shuttering of the X‑ray source [21–23].

Any dose reduction technique usually comes at the cost of increased image noise and artifacts. Adequate image reconstruction techniques can mitigate these drawbacks and are therefore a major component of CT dose reduction.

Filtered back projection (FBP) is an analytical reconstruction algorithm relying on the exact mathematical relation between measured projection and reconstructed image data. The speed and robustness of FBP have made it the workhorse of CT reconstruction for decades [15, 24]; however, the assumption of noise-free data and the amplification of noise by the filter severely limit the quality of FBP-reconstructed CT images. In contrast, iterative reconstruction (IR) techniques can reduce image noise through iterative filtering or close to reality physical modeling of the data acquisition process [15]. IR algorithms can be categorized into three stages. Image domain-based reconstruction was the first clinically approved technique in 2009 and features high reconstruction speed; however, noise reduction is limited due to the rather simple iterative denoising only in image space. Hybrid IR algorithms, such as iDose (Philips Healthcare, Best, The Netherlands), ASIR (GE Healthcare, Milwaukee, WI, USA), or SAFIRE (Siemens Healthineers, Erlangen, Germany), feature increased noise reduction and reconstruction time through iterative filtering of both projection and image data. The last stage is represented by model-based IR algorithms (MBIR), which use advanced models in an iterative process of backward and forward projections. They achieve the highest level of noise reduction and ensuing dose reduction but are also computationally most demanding.

The emergence of artificial intelligence (AI) bears great potential for further dose reduction at almost all stages of CT imaging. On the acquisition side, AI-based algorithms have been developed to automatically position the patient at the isocenter using an infrared camera, select the scan range for the required anatomical coverage, or determine tube parameters, in order to optimize patient exposure [25]. On the reconstruction side, AI can be applied to denoise reconstructed images or even perform the reconstruction itself [15, 25]. One common approach is to train a convolutional neural network (CNN) with (simulated) low dose (LD) or artificially noise-enhanced data to reconstruct standard dose (SD) high-quality CT images [26–29]. The application of a CNN is computationally inexpensive once it is trained and validated, when compared to MBIR algorithms. Besides improved IQ, noise reduction, and artifact reduction, reconstruction speed is thus another benefit of AI-based reconstruction. The use of AI in CT imaging will further reduce the required dose; however, study results usually cannot be readily generalized as AI networks are trained on specific datasets. This lack of generalizability must first be addressed to translate AI-based dose reduction to the patient and the ultimate clinical set-up.

Dose reduction techniques are increasingly being used for spinal CT; however, they have not been systematically reviewed yet. Therefore, we aimed to systematically review dose reduction and clinical applications of LD-CT on the spine. Our objective was to determine the degree of clinically achievable dose reduction and the effects on IQ, diagnostic confidence, and patient outcomes. For this purpose, we focused on four major clinical indications: (i) vertebral fractures (VF) and spinal trauma, (ii) spinal degeneration, (iii) perioperative evaluation and (iv) interventional procedures.

## Material and Methods

### Search Strategy

A search of the online database PubMed (http://www.ncbi.nlm.nih.gov/pubmed) was performed to identify studies evaluating methods to reduce radiation dose for spinal CT with respect to the following four clinical indications: (i) VF and spinal trauma, (ii) spinal degeneration, (iii) perioperative evaluation, and (iv) interventional procedures. Studies on CT dose reduction for evaluation of spinal metastases and inflammation were very scarce and therefore not included. The search was conducted by two persons (radiologists with 7 and 4 years of experience, respectively) without a beginning search date (search end date: 12 April 2022). Uncertainties about inclusion of a respective article, if present, were resolved by consensus through discussion with a third reviewer (board-certified consultant in radiology, 11 years of experience).

The literature search was performed according to the preferred reporting items for systematic reviews and meta-analyses (PRISMA) guidelines [30, 31]. The used search terms for PubMed are available in the appendix.

### Inclusion Criteria

Studies were included if they met the following inclusion criteria: (1) study population: human studies including adult or pediatric patients; (2) study design: retrospective or prospective; (3) indications: diagnostic CT for present or suspected spinal pathology, CT for spinal intervention planning or guidance, or perioperative spinal CT; (4) scanning type: noncontrast and/or contrast-enhanced CT covering the entire spine or parts of the spine; (5) purpose: comparison of LD to SD protocols through CT data acquired at different dose levels, CT data acquired at a single dose level and additionally simulated at different dose levels, or CT data including a dose comparison between patient subgroups.

### Exclusion Criteria

Studies were not considered if they met the following exclusion criteria: (1) article type: case reports, case series, conference abstracts, letters, editorials, reviews, meta-analyses, or surveys; (2) language of publication other than English; (3) studies in cadavers, phantoms, or animals; (4) different acquisition technique (e.g., cone beam CT, fluoroscopy, conventional radiography); (5) studies with other purposes (e.g., comparison of shielding techniques, medical staff radiation exposure report).

### Extraction of Data

The following basic information was extracted: (1) author(s); (2) year of publication; (3) number of subjects (*n*) of the entire study and relevant patient subgroups (e.g., SD group, LD group); (4) scanned spine region, type of intervention (if applicable); (5) details on group comparisons (if applicable); (6) details on the used CT system, including number of detector rows, vendor, and model name; (7) image acquisition parameters; (8) image reconstruction algorithms and parameters; (9) dose reduction (in %) and reported dose values: CT dose index (CTDI_vol_), dose length product (DLP), and/or effective dose (E).

## Results

### Study Selection

**Fig. 1:**
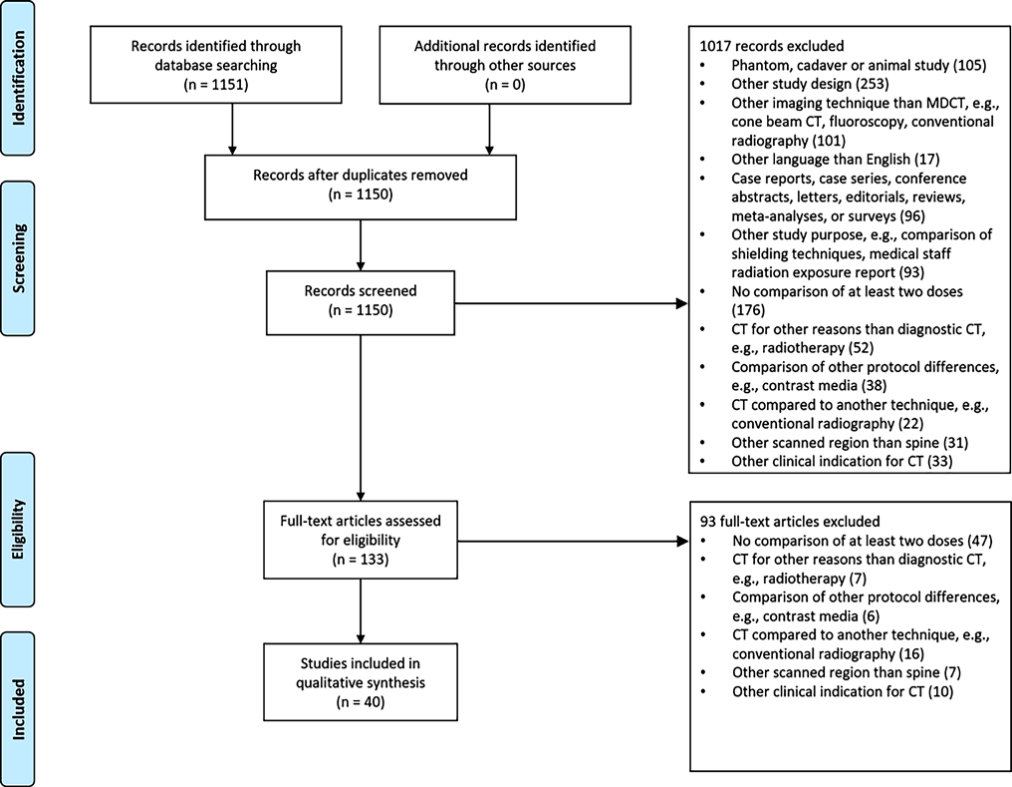
PubMed search flow diagram according to the preferred reporting items for systematic reviews and meta-analyses (PRISMA) guidelines [30, 31]. The used PubMed search terms are available in the appendix

The search via PubMed resulted in 1150 publications after removal of duplicates (Fig. 1). During screening of titles and abstracts, 1017 records were discarded. The assessment of full-text articles led to the removal of 93 records, resulting in 40 publications that were included in the qualitative synthesis for this systematic review.

### Study Characteristics

The 40 selected studies covered VF and spinal trauma (*n* = 14), spinal degeneration (*n* = 6), perioperative evaluation (*n* = 3), and interventional spinal procedures (*n* = 17).

#### Patients

The total number of subjects (*n*) as well as the number of subjects in the SD group(s) and LD group(s) were extracted when provided. Furthermore, the number of included CT examinations or subject numbers for relevant subgroups (e.g., CT scanner, BMI, preoperative/postoperative examination, fracture status, complication status) were extracted for some studies. Total numbers ranged from *n* = 20 [32] to *n* = 380 patients [33] and *n* = 1923 CT examinations [34].

#### Scanned Spine Region

The most frequently covered region by CT imaging was the lumbar spine (*n* = 25), followed by the cervical (*n* = 11), thoracic (*n* = 4), and sacral spine (*n* = 4). Four studies included scans of the whole spine. CT examinations of sacroiliac joints and chest/abdomen/pelvis were counted as sacral spine and thoracic and lumbar spine, respectively.

#### CT System, Acquisition and Reconstruction Parameters

All studies included in this systematic review used multi-detector CT (MDCT). Tube voltages of 75–140 kV were used. In the majority of studies, LD protocols were built upon reduced tube currents, which were determined with different approaches: (i) fixed mA values or ranges, or (ii) reference mA values or ranges in the case of automated tube current modulation. Reporting of mA was heterogeneous, including reference values, mean or median values, and ranges. Statistics on the reported numbers would therefore not be meaningful to present. As an alternative or in addition to tube current, some studies reported tube current-time products, which take into account exposure time. The reported mAs values can be used as a measure of radiation exposure, in particular in CT-guided intervention studies.

Image reconstruction by FBP was reportedly used in 9 studies. Especially more recent studies used IR, which included hybrid IR (HIR), statistical IR (SIR), adaptive SIR (ASIR), and model-based IR (MBIR) (*n* = 21). IR was used to create LD protocols and compared to SD protocols with FBP in six studies [35–40]. Reconstruction technique was not reported in 17 studies. As most of those studies were published in 2017 or earlier, it is reasonable to assume that FBP was used.

### Dose Reporting and Dose Reduction Calculation

Studies reported dose as CTDI_vol_ (*n* = 27), DLP (*n* = 29), and E (*n* = 24). Effective dose (E), commonly regarded as the most appropriate indicator of stochastic radiation risk, is derived by multiplying DLP with a conversion factor for a specific CT examination. Different DLP to E conversion factors were used from published studies, which depend on the scanned spine region, patient age, acquisition parameters, and time of publication. Not all studies used the same conversion factors, which ranged from 0.005 mSv/(mGy*cm) at the thoracolumbar spine to 0.020 mSv/(mGy*cm) at the cervical spine [41–49]. Five studies did not report conversion factors at all. As a result, E and dose reductions based thereon should be compared with caution.

**Fig. 2:**
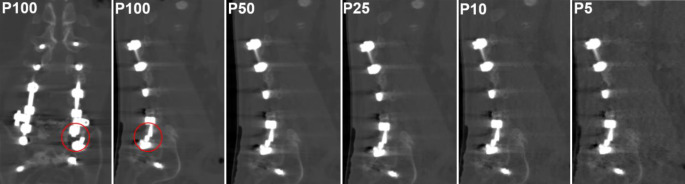
Multidetector computed tomography (MDCT) images of a patient with implant failure after dorsal stabilization using a rod-screw system (spanning L1–S2). Coronal and sagittal MDCT reconstructions with statistical iterative reconstruction (SIR) are shown using 100% of projections (P100), and using sparse sampling with 50% (P50), 25% (P25), 10% (P10), and 5% (P5) of original projections. There is a left-sided rod defect (*red circles*) at the S1 level that is clearly depicted up to a dose reduction of at least 75% (P25)

**Fig. 3:**
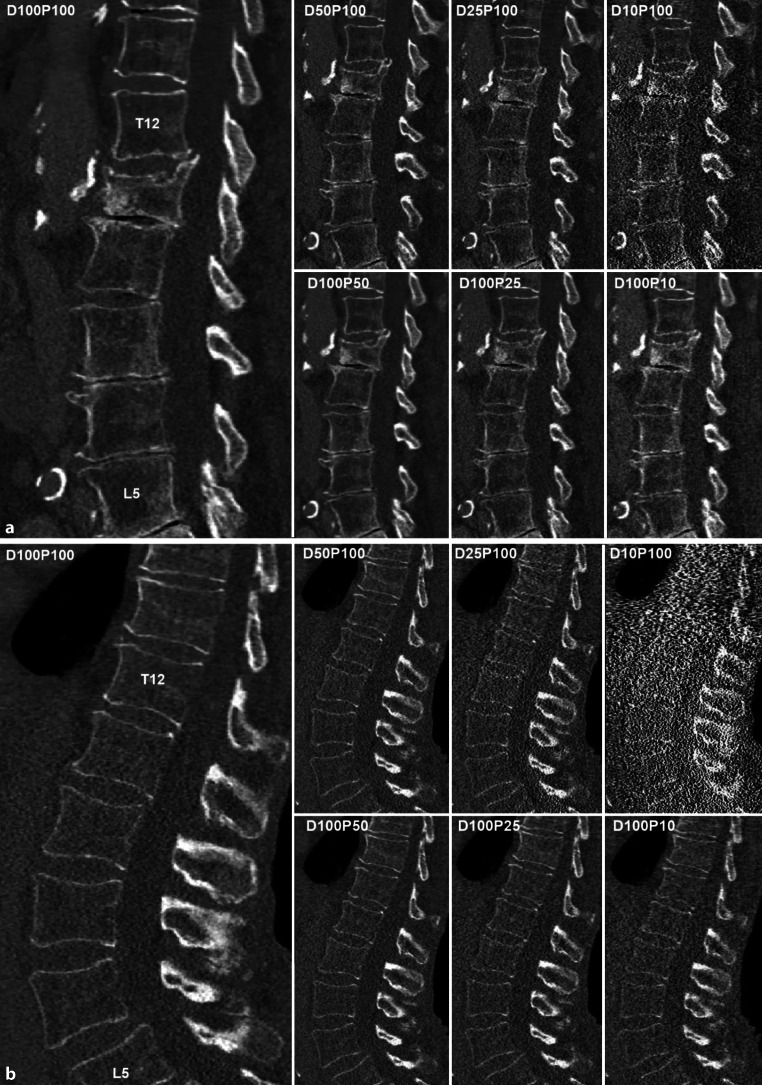
**a** Dose-reduced multidetector computed tomography (MDCT) of the lower thoracic and lumbar spine in a 76-year-old patient with L1 fracture. Sagittal images were reconstructed using statistical iterative reconstruction (SIR) from original MDCT data using (i) full dose and 100% of projections (D100P100), (ii) tube current virtually reduced to 50, 25, and 10% (D50P100, D25P100, D10P100), and (iii) sparse sampling with only every second, fourth, or tenth projection (D100P50, D100P25, D100P10). **b** Dose-reduced multidetector computed tomography (MDCT) of the lumbar spine in a 74-year-old patient without vertebral fractures. Sagittal images were reconstructed using statistical iterative reconstruction (SIR) from original MDCT data using (i) full dose and 100% of projections (D100P100), (ii) tube current virtually reduced to 50, 25, and 10% (D50P100, D25P100, D10P100), and (iii) sparse sampling with only every second, fourth, or tenth projection (D100P50, D100P25, D100P10)

Dose reductions were explicitly reported in 32 studies and retrospectively calculated from provided dose values in 5 studies, 2 studies reported dose reductions based on only CTDI_vol_ [33, 50], 10 studies on only DLP [51–60], 7 studies on only E [34, 61–66], 5 studies on both CTDI_vol_ and DLP [35–39], 1 study on both CTDI_vol_ and E [67], and 1 study on both DLP and E [68]. Dose reduction was retrospectively calculated in one study based on only DLP [69], in two studies on only E [53, 70], and in another two studies on both CTDI_vol_ and DLP [71, 72]. Achieved dose reductions ranged from 6% to 95%, not taking into account simulated LD studies. Simulation of LD data was performed in seven studies, either by virtually lowered tube currents [32, 73], sparse sampling using a reduced number of projections (Fig. 2; [74]), or both (Fig. 3; [75–77]).

Dose values were reported as mean (with or without standard deviation) or median (with or without minimum, maximum, and interquartile range). Only the mean was extracted when mean and median were provided. In Tables 1, 2, 3 and 4, dose values are provided for the SD group, LD group, and subgroups (e.g., scanned region, CT scanner, patient, size, BMI, preoperative/postoperative examination, proceduralist), where reasonably applicable.

**Table 1:** Dose reduction in vertebral fractures and spinal trauma

Author	Year	Subjects (*n*)	Scanned region	Comparison	CT System	Acquisition parameters	Reconstruction (name, level)	Dose reduction	CTDI_vol_ [mGy]	DLP [mGy*cm]	E [mSv]
Heggie [51]	2005	205 75^SD, MSCT^ 74^LD, MSCT^ 56^CD,SSCT^	Lumbar	SD^MSCT^ vs LD^MSCT^ vs CD^SSCT^	SSCT (Siemens Somatom Plus 4) 16-MSCT (Siemens Sensation 16)	120 kVp, 360 mAs ^SD,MSCT^ 120 kVp, 300 mAs ^LD,MSCT^ 140 kVp ^SSCT^	NA	20%	NA	560.0^SD^ 455.0^LD^ 455.0^CD^	6.1^LD^
Mulkens [67]	2007	191 51^SD^ 140^LD^	Cervical	SD vs LD 6‑MDCT vs. 16-MDCT	6‑MDCT (Siemens Emotion 6) 16-MDCT (Siemens Sensation 16)	130 kV, 175 mAs ^SD,6^ 120 kV, 250 mAs ^SD,16^ 110–130 kV ^am^ ^LD,6^ 100–120 kV ^am LD,16^	NA	61–71%	23.2^SD,6^ 19.2^SD,16^ 15.3–23.2^LD,6^ 12.5–19.48^LD,16^	NA	3.8^SD^ 1.1–1.6^LD^
Maxfield [35]	2012	245 109^SD^ 136^LD^	CAP BC	SD^FBP^ vs. LD^ASIR^	64-MDCT (GE Lightspeed VCT)	NA	FBP ASIR	20%^CTDI,DLP^	17.1^SD,CAP^ 14.2^LD,CAP^ 61.7^SD,BC^ 49.6^LD,BC^	1165.0^SD,CAP^ 1004.0^LD,CAP^ 1327.0^SD,BC^ 1067.0^LD,BC^	19.8^SD,CAP^ 17.1^LD,CAP^
Geyer [36]	2013	147 67^SD^ 80^LD^	Cervical	SD^FBP^ vs LD^ASIR^	64-MDCT (GE Lightspeed VCT XT)^SD^ 64-MDCT (GE Discovery HD 750)^LD^	120 kV, max. 300 mAs^am^	FBP ASIR (30%)	55%^CTDI^ 54%^DLP^	21.4^SD^ 9.6^LD^	441.2^SD^ 204.2^LD^	2.4^SD^ 1.1^LD^
Ardley [61]	2013	60 30^rDA^ 30^sDA^ 30^SA^	BC	rDA vs sDA vs SA	128-MDCT (Philips Ingenuity)	120 kV^am^	NA	16%	919.3^sDA,c^ 813.1^rDA,c^	1829.0^rDA,t^ 1735.6^sDA,t^ 1458.7^SA,t^	3.4^SA,t^ 4.0^sDA,t^ 4.2^rDA,t^
Mueck [33]	2014	380 126^SD,STD^ 254^LD,SWIM^	Cervical	SD^SWIM^ vs LD^STD^	64-MDCT (GE Discovery HD 750)	120 kV, 20–300 mA^am^	ASIR (30%)	6%	6.6^SD^ 6.2^LD^	NA	0.8^SD^ 0.7^LD^
Patro [37]	2016	78 48^SD,FBP^ 30^LD,ASIR^	Cervical	SD^FBP^ vs LD^ASIR^	64-MDCT (GE Lightspeed)	120 kV, 100–650 mAs ^SD^ 120 kV, 81–451 mAs ^LD^	FBP ASIR (30%)	36%	16.8^SD^ 10.7^LD^	404.5^SD^ 256.6^LD^	2.4^SD^ 1.5^LD^
Mei [75]	2017	24 12^VF^ 12^nVF^	Thoracic Lumbar	SD vs LD^S1^	256-MDCT (Philips iCT)	120 kV, 200–400 mA, 109 mAs ^SD^ 11–55 mAs ^LD^	SIR	50–90%	7.5^SD^ 0.8–3.8^LD^	NA	NA
Lee [62]	2017	263 126^SD^ 137^LD^	Lumbar	SD vs LD ^BMI^	64-MDCT (Philips Ingenuity)	120 kV, 200–300 mAs^am^ ^SD^ 120 kV, 80–150 mAs^am^ ^LD^	HIR (iDose 4)	47–69%	11.9^SD^ 6.2^LD^	350.5^SD^ 188.4^LD^	4.9^T^, (3.6/4.7/5.7^BMI^) ^SD^ 2.1^T^, (1.1/2.0/3.0^BMI^) ^LD^
Weinrich [63]	2018	80 40^SD^ 40^LD^	Lumbar	SD vs LD	256-MDCT (Philips Brilliance iCT)	120 kV, 158 mAs^r SD^ 140 kV, 70 mAs^r LD^	HIR (iDose 3^SD^, 4^LD^, 6^LD^)	50%^E^	11.4^SD^ 6.9^LD^	403.7^SD^ 209.2^LD^	6.2^SD^ 3.2^LD^
Lee [64]	2018	144 76^SD^ 68^LD^	Lumbar	SD vs LD	320-MDCT (Toshiba Aquilion ONE dynamic volume CT)	120 kVp, 200–300 mAs^am SD^ 120 kVp, 80–150 mAs^am LD^	MBIR (AIDR)	61%^E^	NA	NA	5.4^SD^ 2.1^LD^
Anitha [73]	2019	16 8^VF^ 8^nVF^	Lumbar	SD vs LD^S2^	256-MDCT (Philips iCT)	120 kV, 200–400 mA, 112 mAs ^SD^ 11–56 mAs ^LD^	FBP	50–90%	7.7^SD^ 0.8–3.8^LD^	NA	NA
Sollmann [76]	2019	35 23^VF^ 12^nVF^	Whole spine	SD vs LD^S1^	64-MDCT (Philips Brilliance 64)	120 kV, 143 mA, 180 mAs^am^ ^SD^ 18–90 mAs ^LD^	FBP	50–90%	11.7^SD^ 1.2–5.9^LD^	NA	NA
Tozakidou [65]	2019	68 34^SD^ 34^LD^	Cervical	SD vs LD	128-MDCT (Siemens Somatom Definition AS+)	120 kV, 195 mAs ^SD^ 120 kV, 105 mAs ^LD^	IR (SAFIRE 3)	51%^E^	14.1^SD^ 7.0^LD^	319.7^SD^ 156.4^LD^	1.6^SD^ 0.8^LD^

^am^ automatic tube current modulation, ^BMI^ BMI < 23/23–25/ ≥ 25 kg/m^2, c^ cervical spine dose, ^CD^ comparison dose of SSCT, ^CTDI^ based on CTDI_vol_, ^E^ based on effective dose, ^LD^ low dose, ^nVF^ no vertebral fracture, ^r^ reference mAs value, ^rDA^ retrospective data acquired with dual acquisition technique, ^SA^ single acquisition technique, ^SD^ standard dose, ^sDA^ simulated dual acquisition data acquired with single acquisition technique, ^STD^ standard position, ^SWIM^ swimmer’s position, ^S1^ 10/25/50% of SD using simulated lower tube currents and sparse sampling, ^S2^ 10/25/50% of SD using simulated lower tube currents, ^t^ total dose, ^T^ all subjects, ^VF^ vertebral fracture, ^6^ 6-row-MDCT, ^16^ 16-row-MDCT

*AIDR* adaptive iterative dose reduction, *ASIR* adaptive statistical iterative reconstruction, *BC* cervical spine and brain scan, *CAP* chest, abdomen and pelvis scan, *FBP* filtered back projection, *MBIR* model-based iterative reconstruction, *MDCT* multi-detector CT, *MSCT* multi-slice CT, *NA* not available, *SAFIRE* sinogram-affirmed iterative reconstruction, *SIR* statistical iterative reconstruction, *SSCT* single-slice CT

**Table 2:** Dose reduction in degenerative spine disease

Author	Year	Subjects (*n*)	Scanned region	Comparison	CT System	Acquisition parameters	Reconstruction (name, level)	Dose reduction	CTDIvol [mGy]	DLP [mGy*cm]	E [mSv]
Bohy [81]	2007	60 8/37/15^BMI1^	Lumbar	SD vs LD^S1^	4‑MDCT (Siemens Somatom Volume Zoom)	140 kV, 200/300/400^BMI1^ mAs ^SD^	NA	35–80%	40.0^SD,a^	NA	NA
Yang [38]	2014	164 50^SD^ 58^LD1^ 56^LD2^	Lumbar	SD vs LD1 vs LD2	256-MDCT (Philips Brilliance iCT)	120 kV, 300 mAs^am^ ^SD^ 120 kV, 150 mAs^am^ ^LD1^ 100 kV, 230 mAs^am LD2^	FBP^SD^ HIR (iDose 4)^LD^	36%^CTDI,LD1^ 47%^DLP,LD1^ 60%^CTDI, DLP,LD2^	18.4^SD^ 10.0^LD1^ 7.3^LD2^	587.5^SD^ 312.6^LD1^ 233.2^LD2^	6.5^SD^ 3.4^LD1^ 2.6^LD2^
Yang [39]	2016	113 55^SD^ 58^LD^	Lumbar	SD vs LD^HIR^ vs LD^IMR^	256-MDCT (Philips Brilliance iCT)	120 kV, 262 mAs^am^ ^SD^ 120 kV, 129 mAs^am^ ^LD^	FBP^SD^ HIR (iDose 4)^LD^ MBIR (IMR 1)^LD^	49%	17.7^SD^ 8.7^LD^	580.5^SD^ 283.4^LD^	6.4^SD^ 3.1^LD^
Iyama [40]	2017	34	Lumbar	FBP vs IMR vs HIR	256-MDCT (Philips Brilliance iCT)	120 kV, 127 mA^am^	FBP MBIR (IMR 1) HIR (iDose 4) MRI^m^	NA	15.6	227.8–743.2	NA
Lee [52]	2017	260 143^LD^ 117^ULD^	Lumbar	LD vs ULD ^BMI2^	64-MDCT (Philips Ingenuity)	120 kV, 150 mAs ^LD^ 120 kV, 30 mAs ^ULD^	IR	60–68%	7.7^LD^ 1.9^ULD^	248.4^LD^ 60.5^ULD^	2.9^T,LD^ 1.5/2.5/4.2^BMI2,LD^ 0.7^T,ULD^ 0.6/0.7/0.8^BMI2,ULD^
Sollmann [77]	2021	26	Cervical Lumbosacral	SD vs LD^S2^	128-MDCT (Philips Ingenuity Core)	120/140 kV, 322 mA, 95 (130–314) mAs^am SD^ 6–98 mAs^am LD^	SIR	50–97%	13.8^SD^ 0.4–6.9^LD^	388.9^SD^	NA

^am^ automatic tube current modulation, ^BMI1^ BMI < 22/22–30/ ≥ 30 kg/m^2, BMI2^ BMI < 23/23–25/ ≥ 25 kg/m^2, CTDI^ based on CTDI_vol,_ ^DLP^ based on DLP, ^LD^ low dose, ^LD1^ low dose using 120 kV and 150 mAs, ^LD2^ low dose using 100 kV and 230 mAs, ^m^ as standard of reference, ^S1^ 20/35/50/65% of SD using simulated lower tube currents, ^S2^ 3/5/10/50% of SD using simulated lower tube currents and sparse sampling, ^SD^ standard dose, ^T^ all subjects

^a^corresponding to a standardized body represented by the Monte Carlo model

*FBP* filtered back projection, *HIR* hybrid iterative reconstruction, *IMR* iterative model reconstruction, *IR* iterative reconstruction, *MBIR* model-based iterative reconstruction, *MDCT* multi-detector CT, *MRI* magnetic resonance imaging, *NA* not available, *SIR* statistical iterative reconstruction

**Table 3:** Dose reduction in perioperative evaluation

Author	Year	Subjects (*n*)	Scanned region	Comparison	CT System	Acquisition parameters	Reconstruction (name, level)	Dose reduction	CTDIvol [mGy]	DLP [mGy*cm]	E [mSv]
Abul-Kasim [70]	2008	127^SD^ 113^LD^ 15^CD^	Thoracic Lumbar	SD vs LD vs CD	16-MDCT (Siemens SOMATOM Sensation)	120 kV, 165 mAs ^SD^ 120 kV, 25 mAs ^LD^ 120 kV, 60 mAs ^CD^	NA	95%^E^	10.1^SD^ 0.5^LD^ 13.0^C^	714.0^SD^ 20.8^LD^ 24.0^C^	13.09^SD^ 0.37^LD^ 0.43^C^
Sensakovic [66]	2016	31 17^SD^ 17^LD^	Thoracic Lumbar	SD vs LD	128-MDCT (Philips Ingenuity Core)	NA^SD^ 100 kV, 25/40^BMI^ mAs ^LD^	NA^SD^ HIR (iDose 5)^LD^	84–91%^E^	8.9/13.0 ^BMI,pre,SD^ 14.1/11.9 ^BMI,po,SD^ 1.0/1.6 ^BMI,pre,LD^ 0.1/1.6 ^BMI,po,LD^	402.7/599.6^BMI,pre,SD^ 553.5/429.5 ^BMI,po,SD^ 53.1/74.8 ^BMI,pre,LD^ 46.1/77.8 ^BMI,po,LD^	7.5/10.7 ^BMI,pre,SD^ 10.5/8.1 ^BMI,po,SD^ 1.0/1.4 ^BMI,pre,LD^ 0.9/1.3 ^BMI,po,LD^
Sollmann [74]	2021	38 24^pc^ 14^nc^	Whole spine	SD vs LD^S^	128-MDCT (Philips Ingenuity Core)	120–140 kVp, 288 mA, 148 mAs^am SD^	SIR	50–95%	12.6^SD^ 0.6/1.3/3.2/6.3^S,LD^	NA	NA

^am^ automatic tube current modulation, ^BMI^ BMI </≥ 25 kg/m^2, CD^ comparison dose based on older CT protocol for surgery planning, ^E^ based on effective dose, ^LD^ low dose, ^nc^ no postoperative complications, ^pc^ postoperative complications, ^po^ postoperative, ^pre^ preoperative, ^S^ 5/10/25/50% of SD using simulated sparse sampling, ^SD^ standard dose

*HIR* hybrid iterative reconstruction, *MDCT* multi-detector CT, *NA* not available, *SIR* statistical iterative reconstruction

**Table 4:** Dose reduction in interventional procedures

Author	Year	Subjects (*n*)	Scanned region	Intervention	Comparison	CT System	Acquisition parameters	Reconstruction (name, level)	Dose reduction	CTDI_vol_ [mGy]	DLP [mGy*cm]	E [mSv]
Shepherd [53]	2011	100 50^SD^ 50^LD^	Whole spine	PRI/PI	SD vs LD	64-MDCT (GE Lightspeed VCT)	120 kV, 549^s^/84^p^/84^g^/199^pc^ mA ^SD^ 120 kV, 149^s^/30^p^/50^g^/50^pc^ mA ^LD^	NA	86%^DLP^ 90%^E,c^ 81%^E,l^	NA	1458.0^SD^ 199^LD^	9.7^c,SD^ 17.5^l,SD^ 1.1^c,LD^ 3.3^l,LD^
Schauberger [82]	2012	80	Lumbar	PRI/PI	Proceduralist^pr^ Patient habitus^dia^	16-MDCT (GE Lightspeed)	120 kV, 100–440 mA^am^ ^p^ 120 kV, 66/42/49/80 mA^pr^ ^g^	NA	NA	88/34/79/149^pr^	NA	NA
Artner [34]	2012	1923 1870^SD^ 53^LD^	Lumbar	PRI/PI	SD vs LD	16-MDCT (Siemens SOMATOM Emotion)	130 kV, 120^s^/80^p^/50^g^ mA ^SD^ 80 kV, 100^s^/80^p^/50^g^ mA ^LD^	NA	85%	NA	NA	1.49^SD^ 0.22^LD^
Artner [54]	2012	100 50^SD^ 50^LD^	Lumbar	PRI/PI	SD vs LD	16-MDCT (Siemens SOMATOM Emotion)	130 kV, 120^s^/80^p^/50^g^ mA ^SD^ 80 kV, 100^s^/80^p^/50^g^ mA ^LD^	NA	85%^no^	NA	94.4^SD^ 13.9^LD^	NA^SD^ 0.2^LD^
Artner [55]	2012	65 5^SD,ctg^ 30^LD,ctg^ 30^flg^	Sacral	PRI/PI	SD vs LD	16-MDCT (Siemens SOMATOM Emotion)	130 kV, 120^s^/80^p^/50^g^ mA ^SD,ctg^ 80 kV, 50^g^ mA ^LD,ctg^ 75–80 kV, 60 mA^flg^	NA	94%	NA	76.3^SD,ctg^ 4.6^LD,ctg^ 3.7^fl^	NA
Paik [56]	2014	247 124^SD^ 123^LD^	Lumbar	PRI/PI	SD^hcp^ vs LD^scp^	16-MDCT (GE Brightspeed Elite)	120 kV, 10^s^/50^p^/30^g^ mA ^SD,hcp^ 120 kV, 10^s^/30^p^/30^g^ mA ^SD,scp^	NA	85%	NA	31.8^SD^ 4.9^LD^	0.5^SD^ 0.09^LD^
Shpilberg [71]	2014	64 35^SD^ 29^LD^	Whole spine	Biopsy	SD vs LD	4‑MDCT (Siemens Volume Zoom) 8‑MDCT (GE Lightspeed Ultra)	120 kV, > 200 mAs ^SD^ 80 kV, 40–60 mAs ^LD^	NA	76%^CTDI^ 61%^DLP^	285.2^SD^ 69.5^LD^	1541.0^SD^ 601.5^LD^	NA
Paik [57]	2015	338 163^SD^ 175^LD^	Cervical	PRI/PI	SD^hcp^ vs LD^scp^	16-MDCT (GE Brightspeed Elite)	120 kV, 10^s^/50^p^/40^g^ mA ^SD,hcp^ 120 kV, 10^s^/40^p^/40^g^ mA ^LD,scp^	NA	80%	NA	39.1^SD^ 7.9^LD^	0.5^SD^ 0.1^LD^
Amrhein [58]	2016	80 40^SD^ 40^LD^	Lumbar	PRI/PI	SD vs LD	16-MDCT (GE Lightspeed)	120 kV, 435(100–440)^p^ mA^am^ ^SD^ 120 kV, 68(50–100)^p^ mA ^LD^	NA	78%^DLP,t^	39.1^p,SD^ 4.2^p,LD^	432.1^t,SD^ 313.1^p,SD^ 94.2^t,LD^ 27.9^p,LD^	NA
Greffier [50]	2017	602 162^SD^ 440^LD^	Lumbar	PRI/PI Vertebral expansion Biopsy	SD vs LD	64-MDCT (Siemens SOMATOM Definition AS+)	120 kV, 275^hm^/60^fm^/60^sm^ mAs^r^ ^SD^ 100^hm^/80^fm^/80^sm^ kV, 200^hm^/60^fm^/60^sm^ mAs^r^ ^LD^	FBP	58%^hm^ 72%^fm^ 72%^sm^	18.3^hm,SD^ 9.2^fm,SD^ 5.1^sm,SD^ 7.9^hm,LD^ 2.6^fm,LD^ 1.5^sm,LD^	NA	NA
Elsholtz [68]	2017	85 22^SD^ 63^LD^	Lumbar	PRI/PI	SD^hcp^ vs LD^scp^	80-MDCT (Toshiba Aquilion PRIME)	120 kV, 20^p^/20^g^ mAs ^SD^ 100 kV, 10^p^/5^g^ mAs ^LD^	NA	64%	NA	8.9^SD^ 3.2^LD^	0.048^SD^ 0.014^LD^
Elsholtz [83]	2017	79 183^tp^	Lumbar	PRI/PI	ULD^BMI1^	80-MDCT (Toshiba Aquilion PRIME)	100 kV, 5 mAs	IR (AIDR)	NA	NA	2.4/23/3.4^BMI^	0.05/0.05/0.07^BMI^
Elsholtz [59]	2019	183 101^SD^ 82^LD^	Cervical	PRI/PI	SD^hcp^ vs LD^scp^	64-MDCT (Siemens SOMATOM Definition)^SD^ 80-MDCT (Toshiba Aquilion PRIME)^LD^	100^p^/100^g^ kVp, NA^am,p^/28^g^ mAs ^SD^ 100^p^/80^g^ kVp, 10^p^/5^g^ mAs ^LD^	FBP^SD^ IR (AIDR)^LD^	93%	NA	22.0^p,SD^ 1.7^g,SD^ 24.3^t,SD^ 0.8^p,LD^ 1.0^g,LD^ 1.8^t,LD^	0.14^t,SD^ 0.01^t,LD^
Sollmann [32]	2019	20	Lumbosacral	PRI/PI	SD vs LD^S1^	128-MDCT (Philips Ingenuity Core)	120 kV, 133 mA, 100 mAs ^p SD^ 120 kV, 1–50 mAs ^*p*^ ^SD^	SIR (A) SIR (B)	50–99%	6.5^SD^ 0.07–3.3^LD^	26.0^SD^ 0.3–13.0^LD^	NA
Cordts [72]	2020	64^pro^ 44^pro,SD^ 20^pro,LD^ 13^pat^	Lumbosacral	LP	SD vs LD	128-MDCT (Philips Ingenuity Core) 256-MDCT (Philips Brilliance iCT)	120 kV, 133 mA, 100 mAs ^SD^ 120 kV, 40 mA, 30 mAs ^LD^	HIR (iDose 4)^SD^ MBIR (IMR)^LD^	69%^CTDI^ 83%^DLP^	6.5^SD^ 2.0^LD^	58.0^SD^ 10.0^LD^	NA
Rosiak [69]	2021	65^pro^ 23^pro,SD^ 42^pro,LD^ 18^pat^	Lumbar	LP	SD vs LD	MDCT (Toshiba/Canon Aquilion One)	120 kV, 100 mA ^SD^ 120 kV, 10 mA ^LD^	NA	89%	NA	248.1^SD^ 26.7^LD^	NA
Paprottka [60]	2022	204 102^SD^ 102^LD^	Cervical Lumbosacral	PRI	SD vs LD	128-MDCT (Philips Ingenuity Core)	120 kV, 40 mA, 30 mAs ^SD^ 120 kV, 20–30 mA, 15–20 mAs ^LD^	MBIR (IMR)	34%^p,DLP^	2.0^p,SD^ 1.8^p,LD^	10.2^p,SD^ 6.8^p,LD^	NA

^am^ automatic tube current modulation, ^BMI^ BMI < 25/25–30/ ≥ 30 kg/m^2, c^ cervical spine, ^CTDI^ based on CTDI_vol_, ^ctg^ CT-guided, ^dia^ anterior-posterior diameter subgroups: < 20/20–30/> 30 cm, ^E^ based on effective dose, ^flg^ fluoroscopy-guided, ^fm^ fluoroscopy mode, ^g^ guide phase, ^hcp^ helical CT for planning, ^hm^ helical mode, ^l^ lumbar spine, ^LD^ low dose, ^no^ non-obese patients, ^*p*^ planning phase, ^pat^ number of patients^, pc^ post contrast images, ^pr^ subgroups by performing proceduralist: 2/8/15/15 years of experience, ^pro^ number of procedures, ^r^ reference mAs value, ^S1^ 1/5/10/50% of SD using simulated lower tube currents, ^s^ survey, ^SD^ standard dose, ^scp^ spot CT for planning, ^sl^ scan length, ^sm^ sequential mode, ^t^ total procedure, ^tp^ total number of procedures

*AIDR* adaptive iterative dose reduction, *ASIR* adaptive statistical iterative reconstruction, *FBP* filtered back projection, *HIR* hybrid iterative reconstruction, *IMR* iterative model reconstruction, *IR* iterative reconstruction, *LP* lumbar puncture, *MBIR* model-based iterative reconstruction, *MDCT* multi-detector CT, *NA* not available, *PRI/PI* periradicular infiltration or pain injection, *SIR* statistical iterative reconstruction

### Outcome Measures

#### Quantitative Measures

Quantitative outcome measures included physical metrics of objective image noise and contrast, as well as other quantitative parameters. A total of 16 studies reported on quantitative image noise, as standard deviation of Hounsfield units (HU) measured in a standardized region of interest (ROI) (*n* = 9) or as signal-to-noise ratio (SNR) (*n* = 9). Contrast-to-noise ratio (CNR) was reported in five studies. Other quantitative parameters were reported in 18 studies: bone parameters (bone mineral density, bone fraction, trabecular number, trabecular separation, trabecular thickness, fractal dimension, finite element analysis, FEA-based failure load) for VF and spinal trauma; dural sac cross-sectional area for spinal degeneration; pedicle width and degree of vertebral rotation for perioperative evaluation; and procedure time and number of scans for interventional procedures.

#### Qualitative Measures

Purely quantitative outcome measures are important to enable a comparable IQ assessment [78]; however, more subjective outcome measures are needed to assess the utility of the images at different doses for the clinical application or diagnostic question. The most frequently reported qualitative measure comparable across all included studies were subjective IQ (*n* = 23), containing common subcategories for some studies (overall IQ, overall artifacts, image contrast, sharpness, and depiction of certain spinal structures), followed by subjective utility or confidence for diagnosis or intervention planning (*n* = 12) and subjective image noise (*n* = 4). These measures usually used 3–5-point Likert scales. Furthermore, other application-specific variables were evaluated as outcome measures and are described in the corresponding sections. In 15 studies, qualitative items were rated by 2 or more readers, and interobserver agreement (IOA), assessed by intraclass correlation coefficient (ICC) or Cohen’s kappa, was reported [79, 80]. Although IOA tended to be slightly lower for LD-CT, it remained at least substantial (> 0.6) in most studies. Diagnostic performance for VF status or common degenerative changes was assessed in five studies, reporting classification metrics (accuracy, sensitivity, specificity; *n* = 3) or area under the curve (AUC; *n* = 2).

### Dose Reduction in Vertebral Fractures and Spinal Trauma

Vertebral fractures and spinal trauma were considered in 14 articles, including 7 studies which performed assessment of VF status [62–64, 67, 73, 75, 76], and were primarily performed at the thoracic or lumbar spine (*n* = 6). One study reported on VF status of the cervical spine [67]. Studies without dedicated VF assessment focused on trauma of the cervical spine (*n* = 6). One study from 2005, comparing optimized patient doses in single-slice CT (SSCT) and multi-slice CT (MSCT), was included and only results of lumbar spine scans were extracted [51]. Results are summarized in Table 1.

#### Vertebral Fracture Evaluation

Not taking into account simulated LD protocols, reported dose reductions ranged from 50% to 71% with preserved subjective IQ for VF detection (based on three studies [63, 64, 67]) and no effect on suggested treatment [64]. Reported doses in terms of CTDI_vol_ and DLP ranged from 0.8 to 23.2 mGy and 188.4–403.7 mGy*cm, respectively. The highest dose reduction of up to 71% was reported in 191 patients by Mulkens et al., who compared different SD and LD protocols using 2 different MDCT scanners [62].

The detection of VFs is an important indication of spinal CT. Good diagnostic performance as well as confidence for fracture detection and determination of fracture age were preserved for dose reductions up to 50% (Fig. 3), demonstrating high IOA [62, 64, 76]. Furthermore, the differentiation of patients with and without VF was investigated in four studies. Lee et al. reported sensitivity, specificity, and accuracy ≥ 95% without significant differences between SD and LD [62, 64]. Two simulated LD studies demonstrated that quantitative bone parameters can reliably be assessed in LD-CT and found significant area under the curve (AUC) values in receiver operating characteristics (ROC) analysis, which could particularly benefit osteoporosis patients. Mei et al. reported an AUC of up to 0.9 without significant differences in bone mineral density (BMD) and certain bone microstructure parameters down to 10% of the SD (Fig. 3; [75]). Using FEA-based failure load, Anitha et al. reported an AUC of 0.7 without a significant difference down to 25% of the SD [73].

#### Spinal Trauma

Studies without dedicated VF assessment reported dose reductions ranging from 6% in 380 patients [33] to 55% in 147 patients [36] without a difference in subjective IQ (based on 7 studies [33, 35–37, 51, 61, 65]) and comparable image noise (based on 3 studies [36, 37, 65]). Reported doses in terms of CTDI_vol_ and DLP ranged from 6.2–21.4 mGy and 156.4–560.0 mGy*cm, respectively, not taking into account examinations covering brain and cervical spine or chest, abdomen, and pelvis. In 2005, lumbar spine scans of SSCT and MSCT were compared and it was shown that protocol optimization of the newly introduced CT hardware might reduce dose by 20% to match SSCT levels [51]. Between 2012 and 2016, 3 studies compared ASIR to FBP for image reconstruction in a total of 470 patients, resulting in dose reductions between 20% (without delayed diagnoses or missed injuries) and 55%, underlining the importance of MDCT as the first-line imaging method for spinal trauma [35–37].

Beyond modulation of tube voltage or current, other approaches for dose reduction were investigated. Ardley et al. compared retrospective and simulated dual acquisitions (DA; two single scans) of the brain and cervical spine to a single acquisition (SA) covering both anatomical regions. Due to the elimination of overscanning and overlap of the 2 regions, a total dose reduction of 16% with excellent diagnostic IQ could be achieved [61]. Mueck et al. compared the effect of different arm positions in 380 cervical trauma patients and found improved IQ at a dose reduction of 6% at the cervicothoracic junction for the swimmer’s position with an optimal shoulder girdle angle > 10°, in particular for higher BMI [33]. Also investigating the lower cervical spine, Tozakidou et al. reported that dose can be reduced by 51% without IQ impairment by using an LD protocol in patients without superimposition of C5 and the shoulder girdle [65].

### Dose Reduction in Degenerative Spine Disease

Degenerative spine disease was considered in six articles, including the evaluation of intervertebral discs (IVDs) (*n* = 5 [38, 39, 52, 77, 81]) and other conditions, such as facet joint osteoarthritis, spondylosis, (pseudo)spondylolisthesis, and intervertebral foramen (IVF) narrowing. Patients with low back pain (LBP) were explicitly investigated in two studies [39, 52]. Results are summarized in Table 2.

In the context of IVD evaluation, achieved dose reductions ranged from 35–97%. Using simulated reduced doses at 20–65% of the BMI-adapted tube charge presets, Bohy et al. found no significant effect on identification of bulging IVDs and IVF compromise, while identification of normal IVDs, spinal canal compromise (for ≤ 50% of SD), and herniated IVDs (for ≤ 35% of SD) was impaired. In this study, no explicit patient dose values were obtained; however, a SD of 40.0 mGy corresponding to a standardized body represented by Monte Carlo simulation was reported. The authors concluded that a dose reduction using 65% of SD could be achieved via modification of BMI-adapted tube charge for suspected lumbar disc disease (LDD) [81].

In two studies published in 2014 and 2016, Yang et al. compared FBP-reconstructed SD-CT with IR-reconstructed LD-CT of the lumbar spine and achieved dose reductions of 36–60% [38, 39]. For LD, an intended dose reduction of 50% was realized using two approaches, pure tube current reduction and simultaneous tube voltage and current reduction, which were both combined with HIR. Subjective IQ, SNR, and IOA were equivalent to SD for IVDs and the majority of the other analyzed anatomic regions for the first approach, while SNR, CNR, and IOA were inferior for the second method [38]. In terms of overall diagnostic acceptability, SNR and CNR, LD-CT with knowledge-based iterative model reconstruction (IMR) appeared to be non-inferior to LD-CT with HIR as well as FBP-reconstructed SD-CT. Furthermore, knowledge-based IMR yielded good IOA for IVD conditions [39].

Lee et al. compared LD-CT to ultralow dose (ULD)-CT of the lumbar spine in 260 LBP patients. Despite lower SNR, ULD showed high IOA with respect to IQ and final diagnosis. In non-obese patients, there was no significant difference in diagnostic performance for LDD [52]. Sollmann et al. investigated virtual LD-CT of the cervical and lumbosacral spine by using simulated tube current reduction or a reduced number of acquired projections [77]. Unsurprisingly, subjective IQ and contrast decreased with virtual dose reduction; however, all degenerative changes under investigation could be detected correctly down to 50% of the standard tube current or number of projections. At higher dose reduction (10% of SD), virtual tube current reduction resulted in frequently missed non-calcified disc herniations, in contrast to sparse-sampled LD which still allowed for correct identification of all degenerative changes. Sparse sampling may therefore have higher potential for further dose reduction in the future.

Using magnetic resonance imaging (MRI) as reference standard, Iyama et al. investigated IQ and interobserver reliability of lumbar spinal CT using different reconstruction techniques [40]. Of note, dural sac cross-sectional area was calculated representing a quantitative parameter that was not used in other studies included in this review. Compared to HIR and FBP, IMR demonstrated higher subjective and objective IQ, higher IOA of spinal stenosis, and narrower limits of agreement in Bland-Altman analysis. The reported dose (CTDI_vol_ = 15.6 mGy; DLP = 227.8–743.2 mGy*cm) was in the range of SD values of the other degenerative spine disease studies (CTDI_vol_ = 7.7–18.4 mGy; DLP = 248.4–587.5 mGy*cm).

### Dose Reduction in Perioperative Evaluation

Perioperative evaluation was considered in three articles, including pediatric spinal surgery for adolescent idiopathic scoliosis (AIS) (*n* = 2) and patients with spinal instrumentation (*n* = 1). Results are summarized in Table 3.

In the context of spinal surgery for AIS, the achieved dose reduction range of 84–95% without a relevant impairment of IQ. Abul-Kasim et al. compared 113 LD-CTs before and after surgical correction to SD-CT acquired in 127 trauma patients and sequential CTs acquired for surgery planning in 15 patients and concluded that LD spinal CT allows detailed preoperative planning and postoperative evaluation [70]. In a total of 31 pediatric patients, Sensakovic et al. additionally found that dose can be reduced to the level of 2‑view radiography and depends on patient size and whether the scan is preoperative or postoperative [66].

To investigate the impact of sparse sampling and SIR on metal artifacts, Sollmann et al. applied simulated LD-CT in 38 patients with (*n* = 24) and without complications (*n* = 14) after spinal instrumentation by using 5, 10, 25, 50, or 100% of the acquired projections (P5, P10, P25, P50, P100) [74]. Although overall IQ decreased and artifacts increased with reduced number of projections, all complications were detected for P100, P50, and 25, and diagnostic confidence was high down to P25, and interreader agreement was substantial to almost perfect. The authors concluded that 25% of the original projections might be still sufficient for detection of major instrumentation-related complications, which equals a 75% dose reduction (Fig. 2).

### Dose Reduction in Interventional Procedures

Interventional procedures were considered in 17 articles. The majority focused on PRIs and other pain injections (*n* = 13), mainly performed at the lumbar and cervical spine. Two articles investigated lumbar punctures (LPs) in spinal muscular atrophy (SMA) patients. Other procedures included spine biopsies and vertebral expansions. Specific outcome measures included procedure time, number of acquired scans and technical success, which were reported in 59% (*n* = 10), 53% (*n* = 9), and 53% (*n* = 9) of the studies, respectively. Results are summarized in Table 4.

#### Periradicular Infiltrations and Other Pain Injections

Excluding LD simulations, reported dose reductions ranged from 34% in 204 PRI patients [60] up to 93% in 183 cervical PRI patients [59] and 94% in 65 sacroiliac joint injection patients [55]. The use of LD protocols did not meaningfully affect the rate of complications [54, 55, 58] or patient-reported pain [53, 59, 68].

CT-guided spinal interventions usually comprise different phases, which can include survey images, planning images, guide images during the procedure, and postcontrast images after the procedure, which all contribute to radiation exposure to the patient. Early studies in 2011 and 2012 used reduced tube voltages and currents to achieve very high dose reductions (81–94%) for different procedure phases without affecting technical success [34, 53–55]. Shepherd et al. achieved the major part of the dose reduction during the guide phase, using sequential axial acquisitions with short scan length instead of helical acquisitions [53]. Artner et al. accomplished a high portion of the dose reduction also in the survey and planning phase. Reducing the scanned area of interest in addition to tube voltage and current still provided sufficient IQ for technical success, although achievable dose reduction is more limited in obese patients [34, 54]. For sacroiliac joint injections, the authors replaced the survey image by palpation of anatomical landmarks, which reduced the dose to the levels of fluoroscopy, an alternative method regularly used for certain pain injections [55].

In addition to modified tube settings, the studies by Paik et al. and Elsholtz et al. used a spot scan instead of helical CT for planning, achieving dose reductions of 64–85% for lumbar and 80–93% for cervical injections [56, 57, 59, 68]. Only modifying the planning phase, Amrhein et al. achieved a dose reduction of 78% by reducing scan length and selecting a fixed tube current based on the body diameter of the patient in the survey scan [58].

Using virtually lowered tube currents in 20 PRI patients, Sollmann et al. found sufficient IQ to not affect confidence for intervention planning down to 10% of the SD [32]. After implementation of an LD protocol with tube currents reduced from 40 mA to 20–30 mA, a study in 204 patients observed no relevant difference in IQ or nerve root determination. The reported dose reduction of 34% did not affect the confidence for both planning and performing PRIs at the cervical and lumbosacral spine [60].

Two studies did not perform a dedicated SD to LD comparison; however, it was found that other factors can have a significant effect on tube settings as well as IQ, and hence dose [82]. Patient habitus had a greater influence than the performing interventionalist, which is in line with results from a ULD study that found increased doses only in patients with a BMI ≥ 30 kg/m^2^ [83].

#### Lumbar Punctures in SMA Patients

**Fig. 4:**
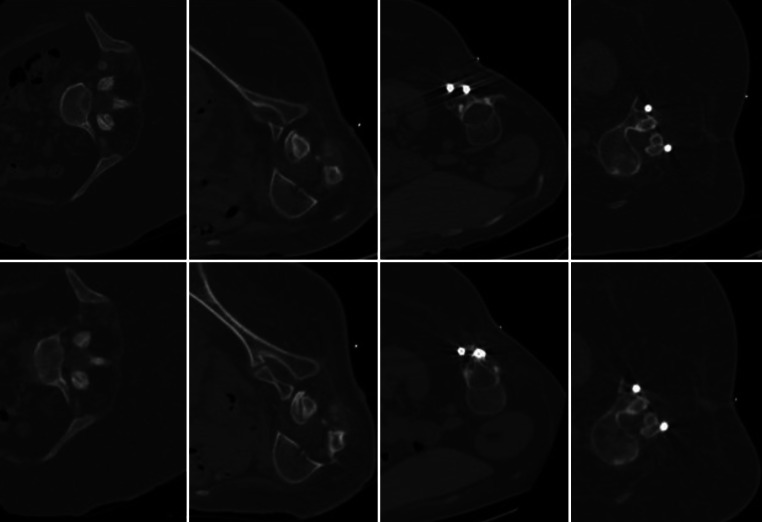
Standard dose (SD; *upper row*) and low dose (LD; *lower row*) multi-detector computed tomography (MDCT) scans for procedure planning of intrathecal nusinersen administration in four patient cases with spinal muscular atrophy (SMA). Tube current was reduced from 133 mA in SD scans to 20, 40, 67 and 27 mA, in LD scans, resulting in considerable dose reductions. The SD scans were reconstructed using hybrid iterative reconstruction (iDose^4^; Philips Healthcare, Best, The Netherlands) while the LD scans were reconstructed using model-based iterative reconstruction (IMR; Philips Healthcare, Best, The Netherlands). The LD images demonstrate a blurrier appearance, but the reduced image quality of the LD scans did not impair the confidence for intervention planning. Due to the model-based iterative reconstruction, the LD images show less streak artifacts from metal implants (third and fourth column)

Intrathecal nusinersen injection is an approved SMA treatment [84]. Patients frequently have severe scoliosis or spondylodesis, requiring CT-guided LP. Since the treatment is performed repeatedly, dose reduction is highly desirable. In 2 studies with 31 patients who underwent a total of 129 procedures dose reductions of 69–89% were found (Fig. 4). The higher dose reduction reported by Rosiak et al. was probably achieved by additional reduction of scan length along the spine [69]. All procedures were successful without increasing procedure time or requiring additional attempts to reach the intrathecal space [69, 72].

#### Other Interventions

CT-guided biopsy is the method of choice for the diagnosis of suspected spinal malignancy. At a dose reduction of 76%, Shpilberg et al. found no difference in the number of scans or procedure time for LD compared to SD protocol. Most importantly, diagnostic tissue yield with respect to malignancy and lesion type (lytic, sclerotic, or mixed) was not affected [71]. Investigating different spinal interventions, Greffier et al. found dose reductions of 58–72%. Highest reductions were achieved with sequential mode and fluoroscopy mode during the guide phase, which therefore should be used instead of helical scanning [50].

### Protocol Recommendations and Recommended Radiation Dose Levels

Methodology and design of the included studies are heterogeneous. Therefore, it is difficult to make universal CT protocol recommendations; however, we derived recommendations for the most important parameters for reduced dose protocols in vertebral fractures and spinal trauma, degenerative spine disease and interventional procedures. For perioperative evaluation, not enough comparable studies were included to derive meaningful protocol recommendations. Table 5 summarizes the derived low dose protocol recommendations. Recommended radiation dose values derived from the studies included in this review were all lower than literature reference values. For comparison, achievable doses (AD) and diagnostic reference levels (DRL), defined as 50th and 75th percentile of recorded radiation doses, respectively, were extracted from [85] and [86]. These dose values are only reported for cervical spine scans and should therefore be referenced with care. Studies published before 2013 were not considered for protocol recommendations.

**Table 5:** Protocol recommendations and reference radiation dose levels

	Tube voltage [kV]	Tube current time product range [mAs]	CTDIvol [mGy]	DLP [mGy*cm]	Reconstruction	Additional considerations
Vertebral fractures and spinal trauma	120	55–105 with ATCM	6–11	204–254	State of the art IR: ASIR, HIR or MBIR	Different arm positions available for cervical spine scans
Degenerative spine disease	120–140	30–150 with ATCM	2–10	61–313	State of the art IR: MBIR better than HIR	Use higher (effective) tube current time product for high BMI patients to maintain sufficient image quality
Interventional procedures	60–120	5–50 without ATCM	2–10^a^	2–94^a^	Use state-of-the-art IR over FBP if available	Optimize acquisition parameters for each phase of the procedure: Survey, planning, guide phase and postcontrast images Spot scanning better than helical scanning for planning images Sequential scanning better than helical scanning for guide phase images Reduce scanned area of interest as much as possible Set acquisition parameters according to patient habitus as scans are usually performed without ATCM
Achievable dose (reference)	–	–	17–25	362–531	–	–
Diagnostic reference level (reference)	–	–	23–33	495–703	–	–

*ASIR* adaptive statistical reconstruction, *ATCM* automatic tube current modulation, *IR* iterative reconstruction, *HIR* hybrid iterative reconstruction, *MBIR* model-based iterative reconstruction

^a^Depending on phase of the procedure

## Discussion

In this article, dose reduction techniques for spinal CT were systematically reviewed. We included 40 studies representing the most common clinical indications. Comparison of LD and SD was most frequently performed between modified tube settings and reconstruction techniques.

For evaluation of VF and spinal trauma, achieved dose reductions ranged from 6–71%. The majority of studies reduced the dose by at least 50% while maintaining overall diagnostic performance and confidence. Besides tube settings and reconstruction techniques, patient positioning and decreasing overlapping scan regions were approaches to reduce exposure. For evaluation of degenerative spine disease, dose reductions without a negative effect on diagnostic performance and acceptable IQ range of 35–50%. Although not consistently investigated across all included studies, overall dose reduction potential tended to be higher for more advanced reconstruction techniques and nonobese patients. Highest dose reductions were achieved for perioperative evaluation and interventions. The reported values for perioperative evaluation ranged from 75% to 95% without negatively affecting the clinical value of the images. For interventional procedures, dose reductions ranged from 34% to 93%, largely depending on the dose reduction approach as well as type and targeted phase of the procedure. The majority of those studies even achieved a dose reduction of > 70% while maintaining sufficient IQ for planning and guidance.

Dose reduction in spinal CT has in a large part been achieved by modifying tube settings while ensuring acceptable IQ using advanced reconstruction techniques. While these advances in LD-CT have been effectively enabled by new software, current and future developments in CT hardware will very likely increase dose reduction. Sparse-sampled CT enabled an additional dose reduction by a factor of 2 or more in simulation studies of the spine [71–74] as well as in other indications [87]. Clinical translation can be expected once the required X‑ray tube technology is available for patient examinations [23]. Up to now, spinal applications of spectral CT have mainly been restricted to artifact reduction [88]. The clinical introduction of photon counting CT (PCCT) can be considered a new era for CT imaging, also with respect to radiation exposure [89, 90]. This innovative technology is expected to further improve IQ mainly due to reduction of electrical noise and artifacts, thus enabling dose reductions. Furthermore, it will potentially advance quantitative capabilities of spinal CT, such as more accurate BMD measurements and bone marrow quantification via material decomposition. Another emerging technique on the brink of clinical translation is AI which can be expected to bring additional dose reductions to spinal CT affecting both acquisition (e.g., via optimized patient positioning or scan volume selection) and reconstruction (e.g., via CNNs trained on low-quality LD and high-quality SD data) [25–29].

In conclusion, considerable dose reduction in spinal CT can be realized by general approaches, such as tube setting modifications and advanced image reconstruction, but can be further increased through specific techniques for certain applications. Additional dose reduction up to 50% with comparable image quality can be expected from the clinical transition of novel acquisition and reconstruction techniques in the upcoming years.

## Appendix

### PubMed Search Terms

#### Dose Reduction in Vertebral Fractures and Spinal Trauma

- ((computed tomography) OR (CT)) AND ((low-dose) OR (low dose) OR (dose reduction) OR (low-kilovolt) OR (low kilovolt) OR (low-kV) OR (low kV) OR (iterative reconstruction)) AND ((vertebral fracture) OR (spinal fracture) OR (vertebral trauma) OR (spinal trauma)).

#### Dose Reduction in Degenerative Spine Disease

- ((computed tomography) OR (CT)) AND ((low-dose) OR (low dose) OR (dose reduction) OR (low-kilovolt) OR (low kilovolt) OR (low-kV) OR (low kV) OR (iterative reconstruction)) AND ((degenerative spine) OR (spinal degeneration) OR (osteochondrosis) OR (spinal stenosis) OR (neuroforaminal stenosis) OR (scoliosis) OR (disc herniation) OR (disc protrusion) OR (degenerative disc disease) OR (facet arthropathy) OR (facet joint arthrosis)).

#### Dose Reduction in Perioperative Evaluation

- ((computed tomography) OR (CT)) AND ((low-dose) OR (low dose) OR (dose reduction) OR (low-kilovolt) OR (low kilovolt) OR (low-kV) OR (low kV) OR (iterative reconstruction)) AND ((postoperative spine) OR (dorsal stabilization) OR (ventral stabilization) OR (spinal instrumentation) OR (vertebral body replacement) OR (intervertebral disc replacement) OR (screw) OR (rod) OR (cage) OR (adjacent segment disease) OR (adjacent segment degeneration)).

#### Dose Reduction in Interventional Procedures

- ((computed tomography) OR (CT)) AND ((low-dose) OR (low dose) OR (dose reduction) OR (low-kilovolt) OR (low kilovolt) OR (low-kV) OR (low kV) OR (iterative reconstruction)) AND ((periradicular infiltration) OR (periradicular therapy) OR (periradicular intervention) OR (PRT) OR (epidural injection) OR (epidural steroid injection) OR (ESI) OR (facet joint infiltration) OR (facet joint therapy) OR (facet joint intervention) OR (facet infiltration) OR (facet therapy) OR (facet intervention) OR (FJI) OR (spinal injection) OR (lumbar puncture) OR (LP) OR (intrathecal administration) OR (intrathecal injection) OR (disc biopsy) OR (vertebral biopsy) OR (vertebral body biopsy) OR (spinal biopsy)).

## Abbreviations

AEC: Automatic exposure control
AI: Artificial intelligence
AIDR: Adaptive iterative dose reduction
AIS: Adolescent idiopathic scoliosis
ASIR: Adaptive statistical iterative reconstruction
AUC: Area under the ROC curve
BC: Cervical spine and brain scan
BMD: Bone mineral density
BMI: Body mass index
CAP: Chest, abdomen and pelvis scan
CNN: Convolutional neural network
CNR: Contrast-to-noise ratio
CT: Computed tomography
CTDI_vol_: Volumetric CT dose index
DA: Dual acquisition
DECT: Dual energy CT
DLP: Dose length product
E: Effective dose
FBP: Filtered back projection
FEA: Finite element analysis
HIR: Hybrid iterative reconstruction
HU: Hounsfield units
ICC: Intraclass correlation coefficient
IMR: Iterative model reconstruction
IOA: Interobserver agreement
IQ: Image quality
IR: Iterative reconstruction
IVD: Intervertebral disc
IVF: Intervertebral foramen
KV: Tube voltage
LBP: Low back pain
LD: Low dose
LD-CT: Low dose CT
LDD: Lumbar disc disease
LP: Lumbar puncture
MA: Tube current
MAs: Tube current-time product
MBIR: Model-based iterative reconstruction
MDCT: Multi-detector CT
MRI: Magnetic resonance imaging
MSCT: Multi-slice CT
NA: Not available
PCCT: Photon counting CT
PRI: Periradicular infiltration
PRISMA: Preferred reporting items for systematic reviews and meta-analyses
ROC: Receiver operating characteristics
ROI: Region of interest
SA: Single acquisition
SAFIRE: Sinogram-affirmed iterative reconstruction
SD: Standard dose
SD-CT: Standard dose CT
SIR: Statistical iterative reconstruction
SMA: Spinal muscular atrophy
SNR: Signal-to-noise ratio
SSCT: Single-slice CT
STD: Standard position
SWIM: Swimmer’s position
ULD: Ultralow dose
ULD-CT: Ultralow dose CT
VF: Vertebral fracture
